# Chopping and Changing: the Evolution of the Flavin-dependent Monooxygenases

**DOI:** 10.1016/j.jmb.2016.07.003

**Published:** 2016-07-31

**Authors:** Maria Laura Mascotti, Maximiliano Juri Ayub, Nicholas Furnham, Janet M. Thornton, Roman A. Laskowski

**Affiliations:** 1 IMIBIO-SL CONICET, Facultad de Química Bioquímica y Farmacia, Universidad Nacional de San Luis, Ejército de los Andes 950, San Luis D5700HHW, Argentina; 2 Department of Pathogen Molecular Biology, London School of Hygiene and Tropical Medicine, Keppel Street, London WC1E 7HT, UK; 3 EMBL-EBI, Wellcome Trust Genome Campus, Hinxton, Cambridge CB10 1SD, UK

**Keywords:** FAD, flavin adenine dinucleotide, FMN, flavin mononucleotide, FMO, flavin monooxygenase, MDA, multidomain architecture, EC, Enzyme Commission, GR, glutathione reductase, PDB, protein data bank, ML, maximum likelihood, BS, bootstrap, BVMO, Baeyer–Villiger monooxygenase, NMO, N-hydroxylating monooxygenases, MAO, monoamine oxidase, PHBH, *p*-hydroxybenzoate hydroxylase, MSA, multiple sequence alignment, flavin-dependent monooxygenases, multidomain architecture, cofactor-binding enzymes, enzyme evolution

## Abstract

Flavin-dependent monooxygenases play a variety of key physiological roles and are also very powerful biotechnological tools. These enzymes have been classified into eight different classes (A–H) based on their sequences and biochemical features. By combining structural and sequence analysis, and phylogenetic inference, we have explored the evolutionary history of classes A, B, E, F, and G and demonstrate that their multidomain architectures reflect their phylogenetic relationships, suggesting that the main evolutionary steps in their divergence are likely to have arisen from the recruitment of different domains. Additionally, the functional divergence within in each class appears to have been the result of other mechanisms such as a complex set of single-point mutations. Our results reinforce the idea that a main constraint on the evolution of cofactor-dependent enzymes is the functional binding of the cofactor. Additionally, a remarkable feature of this family is that the sequence of the key flavin adenine dinucleotide-binding domain is split into at least two parts in all classes studied here. We propose a complex set of evolutionary events that gave rise to the origin of the different classes within this family.

## Highlights

• Changes in domain architectures reflect the phylogeny of flavin monooxygenases.
• Recruitment of different domains has been the main force driving its evolution.
• A notable feature of flavin monooxygenases is that the flavin adenine dinucleotide-binding domain is split.
• Classes of monooxygenases emerged from an ancestral domain by structural changes.

## Graphical Abstract

**Figure:**
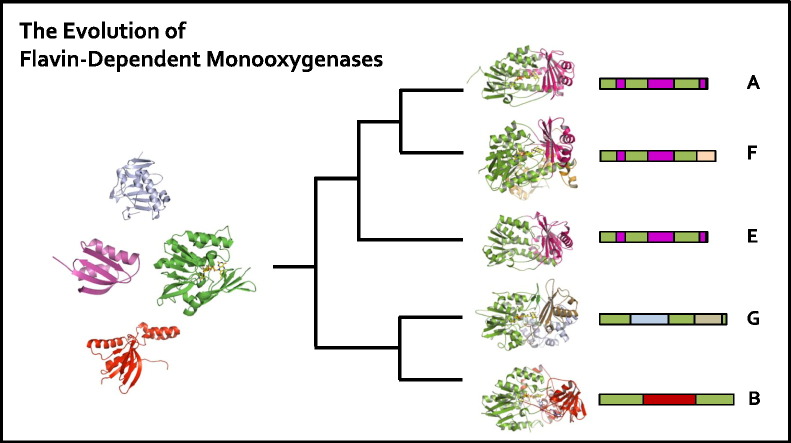

## Introduction

Flavin-dependent enzymes are widespread in nature and perform a wide variety of redox reactions including hydroxylation, reduction, halogenation, monooxygenation, DNA repair, light emission, and cellular signaling [1]. The chemistry underlying these diverse reactions differs from case to case and makes the flavoproteins stand out from most other cofactor-dependent enzymes [2]. There has been much interest in these enzymes as their remarkable selectivity makes them promising tools for biocatalytic applications [3], and a large number have been characterized to date [4], [5].

Their cofactors, the flavins, are versatile redox compounds derived from vitamin B2 (riboflavin), the most common being flavin adenine dinucleotide (FAD) and flavin mononucleotide (FMN). Flavins can receive up to two electrons from a reducing compound and then transfer them to electron acceptors. Two major groups of flavin-dependent enzymes react with molecular oxygen: the flavin-dependent oxidases and the flavin-dependent monooxygenases [6]. The latter are the subject of this study. They react with molecular oxygen to form the activated species C4a-(hydro)peroxyflavin, which is capable of incorporating a single oxygen atom into an organic substrate and of catalysing the hydroxylation, epoxidation, Baeyer–Villiger oxidation, and heteroatom oxidation of a wide range of substrates [7].

Flavin-dependent monooxygenases, which show a high degree of sequence divergence, have been classified into eight classes (A–H) [8], [9]. In terms of mechanism, they fall into two groups: the first consists of classes A [10], B [11], and G [12] in which the reactions with the electron donor and oxygen are carried out by a single protein that tightly binds its flavin cofactor. The second group, classes C–F [13], [14] and H [15], uses an external reductase to reduce the flavin that is then delivered to the monooxygenase protein to perform the oxidative reaction. An additional class, named E(A), which includes epoxidases, resembles class A at the sequence level but displays biochemical features akin to the class E monooxygenases [9].

All flavin-dependent monooxygenases belong to the Enzyme Commission (EC) oxidoreductase sub-subclasses: EC 1.14.13, EC 1.14.14, and EC 1.13.12 (for detailed assignments of each class, refer to Huijbers *et al.* [9]). They play major roles in cellular degradation/detoxification processes, such as the metabolism of amino acids, vitamins, cofactors, and terpenoids [16], [17], and in the transformation of xenobiotic compounds [18]. These processes involve similar chemistry performed by proteins that have significantly diverged and, in some cases, have different 3D folds, providing a beautiful demonstration of how nature has solved the same problem using different pathways. Several studies on the origin and divergence of nucleotide-binding proteins have been published, focusing on classifying a large number of different flavin-dependent enzymes such as alcohol dehydrogenases, flavodoxins, thioredoxins, glutathione reductases (GRs), etc. [19], [20]. An interesting study into the evolution of enzyme function has been performed by Ojha *et al.* [21] in the so-called two dinucleotide-binding domain flavoproteins. All flavoproteins having two dinucleotide-binding domains were analyzed, the conclusion being that the major constraint on their evolution was imposed by both the cofactor and the protein–protein (quaternary) interactions. Among flavin-dependent monooxygenases, the only group belonging to the two dinucleotide-binding domain flavoproteins is class B. Therefore, the question of how the evolutionary diversification of all flavin monooxygenases occurred remains unexplored. Moreover, the specific determinants of the enzyme function of each class remain unknown.

Over evolutionary time, nature has employed a limited number of protein folds to produce a large number of enzymes with different functions [22]. The evolution of a novel function can occur through various mechanisms such as single-point mutations, fusion to other domains, and architectural rearrangements [23], [24]. However, as proteins are thermodynamically stable structures, their evolutionary trajectories are limited to a narrow range of stability [25]. For cofactor-binding proteins, a major constraint is the functional binding of the cofactor [26]. Therefore, the cofactor-binding domain might be expected to remain structurally conserved, and this provides a means of detecting distantly related proteins via their structurally conserved regions [27].

In this work, we have explored the evolutionary relationships between the flavin-dependent monooxygenases, specifically focusing on classes A, B, E, F, and G that share a common structural domain. We suggest how, from a common ancestor, the enzymes diverged into the different classes proposed by Huijbers and coworkers [9]. By integrating data from amino acid sequences, 3D structure, multidomain architecture (MDA), chemistry, and phylogenetic inferences, we have been able to unveil a complex set of evolutionary factors influencing the evolution of this family and leading to its current diversity.

## Results

The flavin-dependent monooxygenases form a large and diverse family of enzymes [9]. They mostly belong to EC sub-subclass 1.14.13, whose definition is: oxidoreductases, acting on paired donors, with incorporation or reduction of molecular oxygen, with NADH or NADPH as one donor, and incorporation of one atom of oxygen into the other donor. This classification is further subdivided into 205 sub-sub-subclasses, of which 60 correspond to flavin monooxygenases. A further five of our enzymes are found within EC 1.13.12 and four within EC 1.14.14 (Table S1). However, there are also a significant number that have not yet been classified by the EC.

We used the CATH domain classification [29]—which has a hierarchy consisting of four major levels: Class, Architecture, Topology (fold family) and Homologous superfamily—to obtain the domain architectures of all flavin monooxygenases (Table S1). Each domain is assigned a “CATH code” consisting of four numbers—one at each level of the hierarchy, much like the EC numbering hierarchy for enzymes. Proteins sharing the same CATH code to the fourth level are predicted to be evolutionarily related and probably share a common ancestor. For the proteins in our dataset with no 3D structure, we used Gene3D to obtain their predicted domain composition to allow us to include them in our analysis of domain architecture. Gene3D makes use of hidden Markov models to assign likely CATH domains to a given protein sequence [30], [31].

The most common domain found in our enzymes was CATH code 3.50.50.60, which is a three-layer ββα sandwich fold responsible for binding FAD or NAD(P)H. In flavin monooxygenases, it occurs in classes A, B, E, F, and G (Table 1), where it exclusively binds FAD. We will refer to this domain as the FAD-binding domain. It is a common fold found in many other enzymes and is widely distributed among many species. It is similar to the Rossmann fold, and indeed, several papers in the literature refer to the class A and B monooxygenases as having a Rossmann fold [8]. However, the CATH classification makes a clear distinction between our FAD-binding domain, CATH superfamily 3.50.50.60, and the Rossmann fold, CATH superfamily 3.40.50.x with its three-layer αβα sandwich fold.

The FAD-binding domain occurs in different contexts in the flavin monooxygenases. In all cases it is split into two or three parts by the insertion of different domains or subdomains; that is, the FAD domain is formed from two noncontiguous regions of the protein sequence that are nevertheless able to maintain the domain's 3D structure and function. In the case of class B monooxygenases, the inserted domain is a second copy of the FAD-binding domain, as will be described later.

This paper focuses on the classes having the FAD-binding domain in common (i.e., A, B, E, F, and G), which we will refer to as Group 1 classes (Table 1). Of the other classes, members of class C belong to the CATH 3.20.20.30 superfamily of FMN-dependent proteins (TIM-barrel fold); class D comprises CATH domains 1.10.540.10 and 2.40.110.10 corresponding to both subunits of butyryl-CoA dehydrogenases, while class H enzymes have a CATH 3.20.20.70 fold, which is also found in type I aldolases (TIM-barrel fold). These other classes appear to be not evolutionary related to the classes we are studying.

To get a preliminary insight into the evolutionary relationships between these enzymes, we compiled a dataset of 196 enzymes from Group 1 on the basis of their assigned EC numbers (Dataset S1). The sequence divergence among these proteins was too high to be able to determine homology, so a comparison of their 3D structures was undertaken. Structure provides a more sensitive measure of shared ancestry as it tends to be better conserved than sequence over evolutionary time [28]. The 3D structures of 29 of the 196 proteins in our dataset were available in the protein data bank (PDB). An examination of these revealed that the key similarities and differences among the proteins principally involve the arrangement of the structural domains of which they are composed.

### Evolutionary relationships among group 1 flavin-dependent monooxygenases

To probe the evolutionary relationships among the Group 1 classes, we derived a phylogenetic tree based on the structural similarities of the proteins in these classes. Using the available 3D structures (Table S2), we performed an all-against-all structure similarity analysis with the help of the fold comparison program PDBeFold [32]. From the resultant 3D alignments and similarity scores, we used the maximum likelihood (ML) inference method implemented through the PhyML 3.0 software to construct phylogenetic trees [33].

**Fig. 1:**
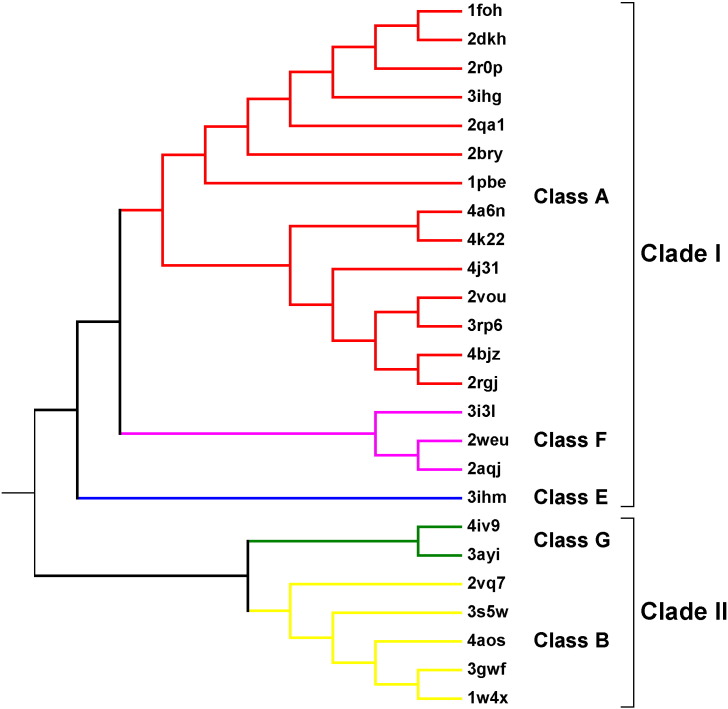
Structure-based phylogeny of the Group 1 flavin-dependent monooxygenases. Molecular phylogenetic analysis by the ML method based on the 3D alignment of the whole 29 available Group 1 protein structures. The PDB codes of each structure are given on the right. The BS consensus tree inferred from 100 replicates was taken to represent the evolutionary history of the taxa analyzed. Branches corresponding to partitions reproduced in fewer than 50% BS replicates are collapsed. The same clade composition and tree topology were obtained when using Bayesian inference. Classes of flavin-dependent monooxygenases are shown in different colors as follows: class A (red), class F (hot pink), class E (blue), class G (green), and class B (yellow).

Figure 1 shows the phylogenetic reconstruction obtained, revealing how the proteins form monophyletic groups according to their class. The tree was rooted, employing a structurally related protein containing the FAD-binding domain. It shows that the structural similarities and dissimilarities reflect the accepted classification. Furthermore, there is a diversification into two major clades: the first containing classes A, F, and E, and the second classes G and B. Interestingly, the topology of the tree does not reflect the chemistry performed by the different enzyme classes. To confirm this, we performed an all-against-all small-structure similarity analysis [34] of the substrates and found that it did not show the same relationships (data not shown) and there was no relationship with the electron donor employed by the classes.

**Fig. 2:**
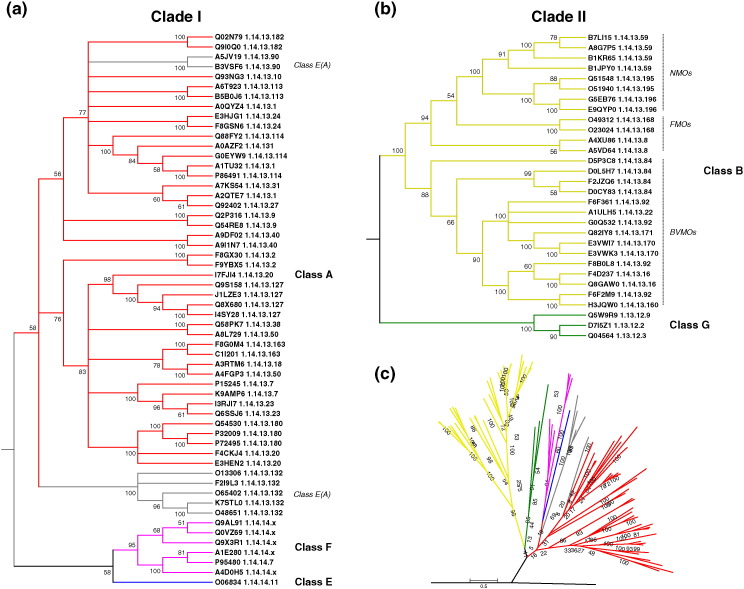
Sequence-based phylogeny of the Group 1 flavin-dependent monooxygenases. Molecular phylogenetic analysis by the ML method from MSAs of manually edited amino acid subsequences corresponding to the FAD-binding domain (CATH 3.50.50.60) only. BS values are indicated at the branches. Branches corresponding to partitions reproduced in fewer than 50% BS replicates are collapsed. UniProt codes and EC numbers are given for each sequence. (a) The phylogenetic tree of the classes included in the first clade of the structure-based tree (Fig. 1). (b) The evolutionary relationships for the second clade in Fig. 1. (c) The phylogenetic tree of all classes of Group 1 flavin‐monooxygenases. The sequence of precorrin synthase (UniProt code: D5AUZ5) was used as an external group to root the tree. Classes of flavin-dependent monooxygenases are shown in different colors: class A (red), class E(A) (grey), class E (blue), class F (hot pink), class B (ochre), and class G (green).

We then focused on the FAD-binding domain common to our five Group 1 classes. Using the sequence similarities of just these domains (from proteins with and without 3D structures), we obtained the phylogenetic tree shown in Fig. 2. Its general topology is consistent with the structure-based tree. However, due to the large divergence of the sequences, some branches display poor statistical support in terms of low bootstrap (BS) values. Thus, further independent, sequence-based phylogenetic analyses were performed for the two main clades in Fig. 1. In the tree containing the Clade 1 classes A, F, and E (Fig. 2a), it is clear that the so-called E(A) class is more closely related to class A than to class E. Moreover, enzymes squalene epoxidase [35] (UniProt accession codes: O13306, F2I9L3, O66402, K7STL0, and O48651) and zeaxanthin epoxidase [36] (UniProt entries: B3V5F6 and A5JV19) do not cluster together, suggesting they diverged independently. These observations indicate that the Class E(A) category should be discarded and its members assigned to Class A. Also, the phylogenetic tree suggests that class F monooxygenases have a closer class-A-like ancestor. The general distribution does not correspond to either the EC classification or the structures of the substrates. The same goes for classes B and G (Fig. 2b). Class B has been reported to include three groups of enzymes; the Baeyer–Villiger monooxygenases (BVMOs), N-hydroxylating monooxygenases (NMOs), and flavin monooxygenases (FMOs) [37]. The phylogenetic relationship within this class is consistent with that previously reported [38]; the BVMOs appear to have diverged from the common ancestor at a different stage than the NMOs, and both form monophyletic groups while the FMOs form a polyphyletic group.

### Domain architecture analyses

**Fig. 3:**
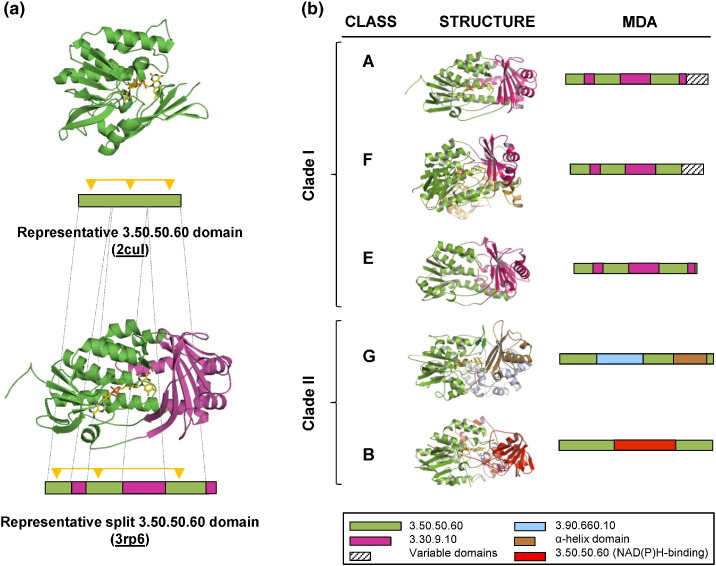
An overview of the MDAs in Group 1 flavin-dependent monooxygenases. (a) Structure and schematic MDA bar diagrams for: Top, a single-domain enzyme consisting of a complete CATH 3.50.50.60 domain (PDB entry: 2CUL); and Bottom, a flavin monooxygenase (3RP6) containing a split 3.50.50.60 domain (green) merged with a split domain 3.30.9.10 (purple). The dashes indicate where the boundaries of the split domain occur in the full domain. The FAD contact sites are represented by the yellow triangles. (b) Overview of the MDAs of each class of flavin-dependent enzymes. Colors in the structure correspond to those in the MDA diagrams. The key at the bottom shows the color code for each CATH domain. Note that the green and purple domains are each a single domain that has been split into segments of noncontiguous amino acids.

A striking feature of our Group 1 flavin-dependent monooxygenases is that, as mentioned previously, the sequence of the FAD-binding domain is split into at least two noncontiguous segments. Figure 3a shows an example. The upper image shows an oxidoreductase protein—a glucose-inhibited division protein A (PDB entry: 2CUL)—consisting of just a single, unsplit FAD-binding domain. The lower image shows one of our Group 1 proteins (PDB entry: 3RP6) containing the same domain in green, but it is split by the intrusion of a different domain, shown in purple. Split domains are observed in about 10% of the protein sequences in the Gene3D database. About 5% of these split domain sequences contain our FAD-binding domain (CATH 3.50.50.60). Interestingly, these split FAD-binding domains are distributed throughout the whole tree of life.

A closer look at Fig. 3b suggests that the divergence into the five different classes of Group 1 is linked to changes in MDA. The enzymes in the first clade (classes A, F, and E) all have a similar architecture consisting of the FAD-binding domain (green) split into three parts of different lengths—of around 70, 80, and 90–100 aa—and merged with CATH domain 3.30.9.10 (purple), which is also split into three parts of around 30, 90–100, and 20 aa. The latter domain is described as a two-layer sandwich and is also found in D-amino acid oxidases [39]. Some Class A monooxygenases also differ at the C terminus, with either the addition of a phenol hydroxylase domain (CATH 3.40.30.20, length: 200 aa) or the presence of unclassified C-terminal extensions of 100 to 150 aa. Moreover, these extensions account for the divergence of class A into the two subclades observed in Fig. 1 (Fig. S1).

In the second clade (classes G and B), major differences are observed. In Class G, the FAD-binding domain is also split into three parts—with lengths of around 120, 90, and 70 aa—but they are interspersed by CATH domain 3.90.660.10 here (light blue, 150 aa), which is typical of subunit A of the monoamine oxidases (MAOs). Additionally, the C-terminal region contains an α-helix domain (brown) of ~ 130 aa [40]. In class B, the domain architecture consists of two copies of CATH domain 3.50.50.60; one of which binds FAD and is split into two halves of 150–180 aa in length (green) and the second (red, and slightly truncated to ~ 100 aa in FMOs and 160 in BVMOs/NMOs) is inserted between them. This second domain tightly binds NADPH instead of FAD, a hallmark of the class B enzymes [9].

The above observations seem to suggest that the divergence of the Group 1 flavin-dependent monooxygenases into the five classes was principally driven at different evolutionary times by the divergence of their domain architectures (Fig. 3b).

### Structural analysis

**Fig. 4:**
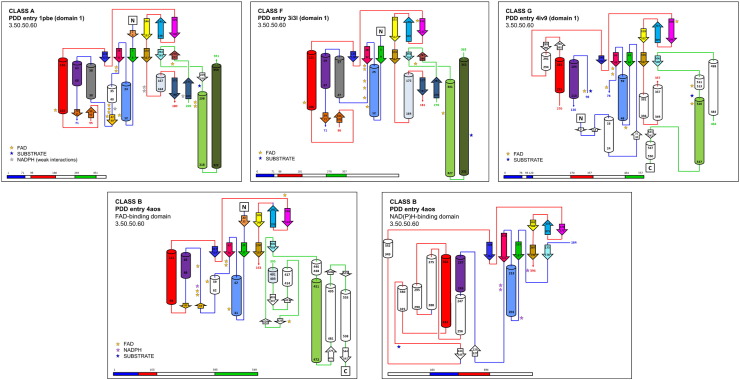
Topology diagrams of the FAD-binding domain. Topology diagrams of the 3.50.50.60 CATH domain in each of the Group 1 classes. Helices are depicted as cylinders and β-strands as arrows. The colors represent secondary structure elements that overlap when the 3D structures are superposed. White elements indicate unique features to the given structure. The stars identify residues involved in the interaction with FAD, NAPH, and substrate. The location of each domain in the protein's MDA is highlighted in the schematic bar diagrams at the bottom. The colors of the bars correspond to the colors of the connecting lines joining the secondary structure elements in the corresponding topology diagram.

As all the 3.50.50.60 domains in these enzymes are split (apart from the second, inserted domain in class B), we examined the locations of the splits and any differences in lost/retained secondary structure elements. Figure 4 shows schematic topology diagrams of the domains (with two for class B). Helices are depicted as cylinders and β-strands as arrows; the latter was arranged according to the β-sheets they form. The coloring reflects common secondary structure elements that coincide when the 3D structures of the domains are superposed. White elements indicate features unique to the given structure. The stars identify residues involved in the interaction with the cofactor and substrate. The most similar diagrams are those of classes A and F. Class G seems to most closely resemble the FAD-binding domain of class B. The internal NAD(P)H-binding domain of class B only shares elements with the first half of the FAD-binding domain, suggesting that it may be the result of a partial duplication combined with an insertion process.

Considering the monophyly of classes A and B and that both are able to use FAD and NAD(P)H, further studies were performed to understand the biochemical differences on the basis of their evolutionary histories.

#### Class A monooxygenases: single nucleotide-binding domain, two bound cofactors

**Fig. 5:**
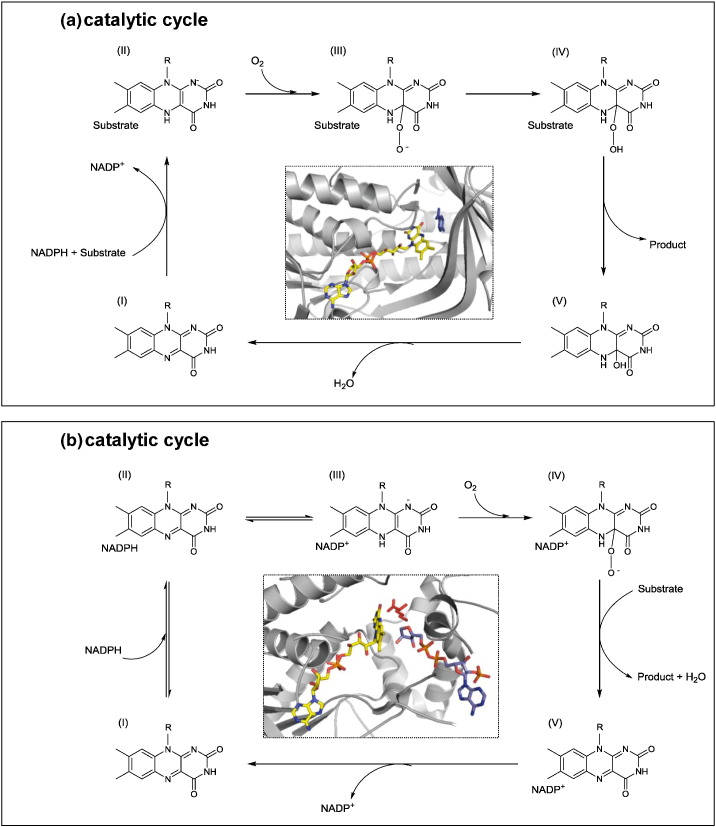
Catalytic mechanisms of class A and class B monooxygenases. Top: schematic representation of the catalytic cycle of class A monooxygenases. The cycle initiates when the ES complex is formed (I) and the oxidized flavin is reduced by NADPH, then the reaction with molecular oxygen takes place to form the C4a-peroxyflavin and C4a-flavin hydroperoxide species (III and IV, respectively); finally, the substrate is oxidized and the product released. At the center, the structure of the active site of PHBH enzyme (PDB entry: 1PBE) is shown, FAD is presented as yellow sticks and substrate *p*-hydroxybenzoate in dark blue. Bottom: as above but for class B monooygenases. Catalytic cycle starts with the recruitment of NADPH (I → II) and the subsequent flavin reduction (III). Reduced enzyme reacts with molecular oxygen to form the key intermediate C4-a peroxyflavin (IV), which then transforms the substrate into the product. NADP^+^ remains bound during the whole catalytic cycle and the rate-determining step in the releasing of this oxidized cofactor (V → I). In the center, the structure of active site of enzyme ONMO is shown (PDB entry: 3S5W), FAD is presented as yellow sticks, NADPH in light blue, and substrate L-ornithine in red.

Class A is the archetypical flavin-dependent monooxygenases [10]. The model enzyme is *p*-hydroxybenzoate hydroxylase (PHBH) [41] (Fig. 5). It has been evidenced that the binding of the nicotinamide cofactor occurs in an extended conformation at the enzyme surface in a groove spanning the FAD-binding site [42], [43]. The NADPH assumes a folded conformation that brings the nicotinamide closer to the isoalloxazine moiety of the flavin during the reduction step. The residues involved in binding NADPH all lie in the FAD-binding domain, indicating that this domain can bind both cofactors but in very different conformations. However, the same residue positions are found in classes F and E, which do not bind a nicotinamide cofactor [13], [44]. Therefore, it seems that class A monooxygenases acquired the ability to bind NADPH after divergence from the other classes by the occurrence of a complex set of changes that formed the groove in the protein's surface (Fig. S2).

The other domain in the class A enzymes is the split 3.30.9.10 domain. It is difficult to envisage how two separate domains might have merged in this way, where each is separately split. A similar domain architecture is found in the D-amino acid oxidases, such as PDB entry: 1C0P [39], where a FAD-binding Rossmann fold domain (CATH 3.40.50.720) also appears to have merged with a 3.30.9.10 domain in a similar way. In this case, the latter domain is involved in defining the substrate cavity. Indeed, in PHBH and other class A monooxygenases, the 3.30.9.10 domain is involved in forming the substrate binding site and in keeping the FAD-binding domain in the proper conformation for binding the flavin cofactor [45]. We will return to the question of merged domains in the Discussion.

Other class A monooxygenases, such as *m*-hydroxybenzoate hydroxylase (PDB entry: 2DKH) and phenol hydroxylase (PDB entry: 1FOH), contain an extra C-terminal domain (CATH 3.40.30.20), which seems to be involved in protein–protein interactions and not in defining the catalytic site. This is also observed for the enzymes angucycline synthase (PgaE; PDB entry: 2QA1), aklavinone 11-hydroxylase (PDB entry: 3IHG), and rebeccamycin synthase (RebC; PDB entry: 2R0P), which have C-terminal extensions that seem to be involved in quaternary interactions; these enzymes take part of multienzyme complexes such as the polyketide synthases [46] (Fig. S1).

#### Class B monooxygenases: two nucleotide-binding domains, two bound cofactors

**Fig. 6:**
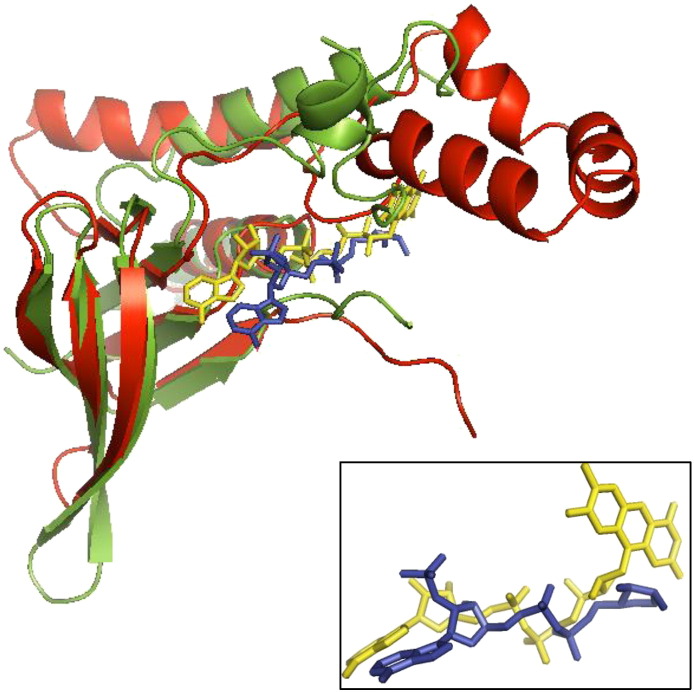
Class B nucleotide-binding domains. Superposition of the first half of a split 3.50.50.60 CATH domain (green) involved in binding FAD (yellow sticks) and an unsplit 3.50.50.60 CATH domain (red) involved in NADPH binding (light blue sticks). Both domains come from PDB entry 3S5W. The RMSD between equivalent Cα atoms is 2.26 Å. The inset at bottom right shows just the cofactors FAD and NADPH from the superposition, demonstrating that they both bind in the same equivalent position and orientation in their respective domain.

The unique domain architecture observed in class B, where a FAD-binding domain has been split in two by the insertion of a slightly truncated copy of the same domain, suggests a duplication event as the origin of this class. The first domain retained its ability to bind FAD, while the second evolved the ability to bind NAD(P)H. Superposition of the two domains places the two cofactors in the same but slightly shifted orientationrelative to one another. From the topology diagram (Fig. 4), it was observed that the NAD(P)H-binding domain resembles only the first half of the FAD-binding domain. When only the two common parts are superposed, the location of the cofactors overlap near exactly (Fig. 6). However, as expected, the residues interacting with the cofactors are not conserved between the two domains. We hypothesize that following the partial duplication of the original domain, mutations in the key residues of the duplicated domain would have been required to change its cofactor specificity as a neofunctionalization process. Indeed, most of the key FAD-binding residues are found in the first, and consequently duplicated, half of the original domain; only two binding residues are located in its second half. Therefore, the partial duplication of the first 160 residues of the original domain was sufficient to produce a second domain capable of binding a nucleotide cofactor.

Of the three kinds of class B monooxygenases, the FMOs have a slightly shorter NAD(P)H-binding domain than the BVMOs and NMOs. The divergence of these three groups could have been the result of changes linked to the structure of the NAD(P)H-binding domain. The FAD-binding domains are virtually identical, in terms of 3D structure, within the class, while the NAD(P)H domains have extra embellishments, such as long interleaved α-helix structures in the BVMOs. In NMOs, the domain remains almost identical to the FAD-binding domain. It is evident that this domain has suffered several structural changes on its surface, while the core involved in the binding of the cofactor has remained conserved.

### The origin of enzyme function in flavin-dependent monooxygenases

**Fig. 7:**
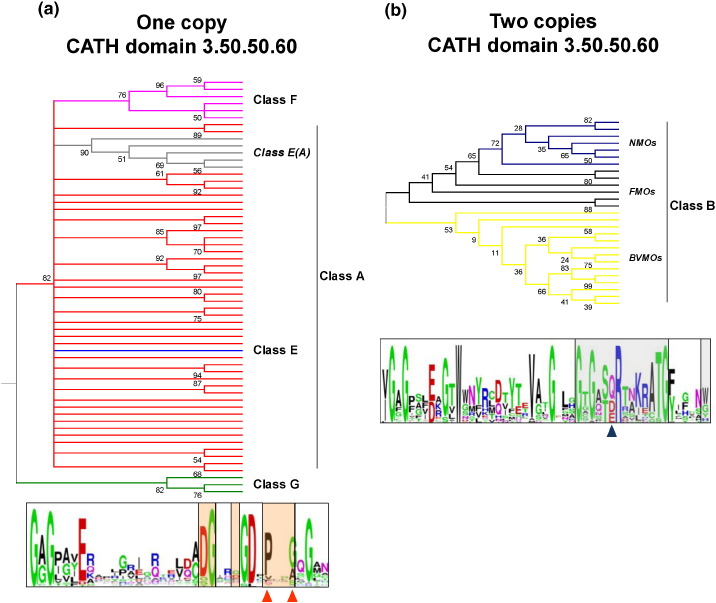
The origin of enzyme function in flavin-dependent monooxygenases. (a) Top: phylogenetic tree of flavin monooxygenases containing a single CATH 3.50.50.60 domain (FAD-binding domain). The tree was constructed from an MSA of subsequences corresponding to just the 39 residues involved in interactions with cofactors and substrates. The ML inference method was used, and BS values are indicated at the branches. Branches corresponding to fewer than 50% BS replicates are collapsed. Colored branches show the different flavin monooxygenases classes: class A (red), class E(A) (grey), class E (blue), class F (hot pink), and class G (green). Bottom: logo scheme employed for the MSA; residues in contact with FAD (empty boxes), with substrate (light orange boxes), and residues in contact with both FAD and the substrate are indicated by orange triangles. (b) Top: as above but for class B enzymes that contain two CATH 3.50.50.60 domains (FAD- and NADPH-binding domains). Here, the colored branches indicate the different subtypes within class B: BVMOs (ochre), NMOs (dark blue), and FMOs (black). Bottom: logo scheme employed for the MSA; residues in contact with FAD (empty boxes) andNAD(P)H (grey boxes), while residues in contact with FAD and NAD(P)H are identified by a blue triangle.

Finally, we examined how the residues involved in binding have changed over evolutionary time and how this might have resulted in the enzymes' diverse functions. We first divided the proteins into two groups: those that have one copy of the FAD-binding domain (classes A, F, E, and G) and those that have two, that is, class B. We then used the SAS (Sequence Annotated by Structure) server [47] to identify the residues interacting with ligands (i.e., cofactors and substrates). This gave us a short subsequence for each protein, and these mini sequences were aligned and compared using multiple sequence alignments (MSAs). The first set of enzymes, having just one copy of the FAD-binding domain, gave 39 interacting residues. Notably, the phylogenetic analyses obtained from their alignment showed that these residues were enough to infer the phylogeny of these classes (Fig. 7a). Classes F and G formed monophyletic clusters, while class A formed a paraphyletic group giving origin to classes F and E. Interestingly, in this tree, the class E(A) monooxygenases are clustered together, indicating that they share common residues that could be linked to their epoxidation activity.

The second group of enzymes, the class B monooxygenases, gave 49 interacting residues. Phylogenetic analysis revealed that these residues contain the information determining their divergence into FMOs, BVMOs, and NMOs (Fig. 7b).

These results evidence the complex set of factors defining and constraining the evolution of flavin-dependent monooxygenases. As proposed above, it seems the main topology of these enzymes' phylogeny has been driven by changes in domain architecture, while the functionalization within the different classes has been driven by the accumulation of changes in the cofactor and substrate-binding sites.

## Discussion

Enzyme evolution is extraordinarily complex, with many different evolutionary events yielding dissimilar chemistries and mechanisms within a superfamily [48]. Understanding those events may help us design new and more efficient enzymes with specific biocatalytic purposes and help predict the function of unknown sequences [49]. Moreover, this knowledge enriches the field of evolutionary biochemistry, providing an understanding of the mechanisms and events that shaped the evolutionary history of organisms [50].

Flavin-dependent monooxygenases are enzymes that display a remarkable versatility in their cellular functions. Here, we have demonstrated that a subset of these enzymes (classes A, B, E, F, and G) shares a common origin and that a complex set of events has taken place during their evolutionary history to result in the differences between them. Their common ancestor would appear to have been a single-domain protein consisting of just a FAD-binding domain. This is a very common, widely distributed fold found in an enormous variety of enzymes [51]. Recently, it has been estimated that it appeared around 2.9 billion years ago at the beginning of the planet's oxygenation, in coincidence with the emergence of aerobic metabolism [52].

**Fig. 8:**
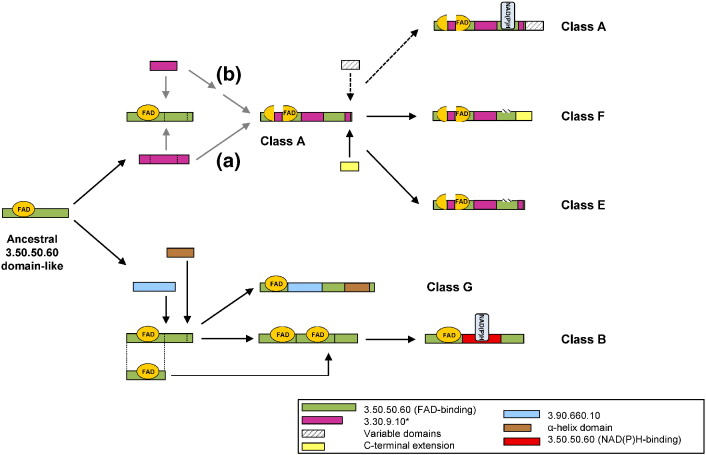
Graphical representation of the proposed evolution and divergence of flavin-dependent monooxygenases. The figure shows the proposed evolutionary events at the MDA level that led from a common FAD-binding ancestor to the emergence of the five classes A, B, E, F, and G of the flavin monooxygenases. Two main divergence events are represented: Top, the fusion to the 3.30.9.10 CATH domain, and the concomitant split of the 3.50.50.60 CATH domain originated classes A, F, and E, alternative (a). The asterisked domain (CATH 3.30.9.10), which, according to Gene3D, is a split domain, is more likely to consist of a single 3.30.70.100 domain at the center and two smaller embellishments, corresponding to the outer parts (see main text). This alternative scenario is presented as (b). Class A is proposed to acquire the ability of NADPH binding after a complex set of structural changes. This class may present an extra C-terminal variable domain (indicated by the dashed arrows). Class F emerged after the recruitment of a C-terminal terminal extension, while class E seems to remain similar to the original common structure. These last two classes lack the ability of binding NADPH. Bottom, class G monooxygenases originated after the recruitment of a CATH domain 3.90.660.10 (involved in substrate binding) and a C-terminal α-helix with the concomitant split of the CATH domain 3.50.50.60 into three parts. On the other hand, class B emerged after a sequence of events involving the partial duplication of the FAD-binding domain and the insertion of this duplicated domain into the original one followed by a change in its specificity toward the binding of NADPH. The inset shows the color code for each CATH domain.

Different evolutionary events led to the divergence of the ancestral and single-domain protein into the five classes. Probably, the divergence into clades I and II (Fig. 1) was the result of one or more fusion events involving the components of the CATH 3.30.9.10 domain to form the substrate-binding pocket and led to the emergence of classes A, F, and E (Fig. 8). In fact, it is unlikely to have been a single fusion event as this would have required the interdigitation of two separate domains. It seems more likely that the insertions happened one at a time and then formed a full domain. Indeed, the definition of the CATH 3.30.9.10 domain is a little spurious, as it only exists in the CATH database in split form—that is, it is always made of separate subdomains. If one considers the insertions one by one, a more likely sequence of events emerges. The largest insertion in Class A enzymes is the middle one of the three purple fragments in Fig. 3b. This can be represented by residues 173–269 of PDB entry 3RP6. A PDBeFold search hits a large number of single-domain proteins (CATH 3.30.70.100), including a number of antibiotic biosynthesis monooxygenases (e.g., PDB entry: 4HL9), so this apparent subdomain of CATH 3.30.9.10 is actually a domain in its own right. The other two insertions—a beta-hairpin unit and a single alpha-helix— are very small fragments, or rather embellishments, that might have been inserted into the FAD-binding domain or evolved by decoration at different evolutionary times.

The recruitment of domains has previously been reported as responsible for the different functionalities in other superfamilies—for example, the Ntn-type amide hydrolase superfamily [53], the ubiquitous haloalkanoate dehalogenase superfamily [54], the ligases [55], and the amazing example of the thiamine-pyrophosphate-dependent enzymes [56]. A remarkable feature of the original FAD-binding domain is that evolution has split it into separate sequence fragments while retaining the 3D fold structure and the ability to bind the flavin cofactor in the correct orientation—that is, with the isoalloxazine ring facing the substrate-binding site. An interesting difference between the classes is that class A monooxygenases can work with both FAD and NAD(P)H cofactors, whereas classes F and E, which share the same MDA, need an external partner to reduce the flavin [13], [44]. A detailed analysis of the residues involved in the binding of the nicotinamide cofactor reveals the difference lies in the appearance of a groove in the surface of class A monooxygenases, which is absent in classes F and E. In Class A, flavin moves rapidly between conformations in the oxidized state, and this mobility has been proposed to be crucial in the transient interaction with NADPH [57]. On the other hand, classes E and F have solved this by accepting exclusively the reduced FAD as a diffusible substrate provided by a partner [6], [10].

A separate set of evolutionary events led to classes G and B (Fig. 8). In the case of class G, the combination with a MAO domain (CATH 3.90.660.10; light blue in Fig. 8) and the fusion of an α-helix domain (brown) gave rise to enzymes with a double function working as both L-amino acid oxidases (oxidative deamination) and monooxygenases (oxidative decarboxylation). The phylogenetic analysis of these enzymes reveals that these proteins are related to the classic MAOs and to the flavin-dependent monooxygenases [12]. For class B, a very different scenario took place. The most plausible explanation of the evidence found in this work proposes that an incomplete duplication event of the FAD-binding domain occurred, combined with the insertion of this partially duplicated domain into the original. Once again, the main constraint during this process would be that the original FAD-binding domain should retain its fold in order to bind the flavin cofactor, regardless of the split. Presumably, when this architecture first formed, the two flavin-cofactor-binding sites may have been subjected to different selective pressures, thus allowing a change in the cofactor specificity of the inserted domain to allow it to bind the nicotinamide nucleotide [58]. A very similar mechanism was proposed some time ago for the GR [59] and other related flavoenzymes [60]. GR contains a FAD-binding domain; thus, possibly, this module was duplicated, fused, and modified to form the NADPH-binding domain. This mechanism seems the most parsimonious since other NADPH-binding domains [20] observed in different enzymes are dissimilar to that observed in GR [61]. It has been reported that superfolds are tolerant to relatively large domain insertions when followed by accommodating mutations in the scaffold, as is observed in the haloalkanoic dehalogenase superfamily [62]. Therefore, in the flavin-dependent monooxygenase family, this step can be considered the origin of class B monooxygenases, which further diverged into different groups according to their specific biochemistry and catalyzed reactions. In virtually all superfamilies, depending on cofactor binding [26], the regions of the protein involved in cofactor binding are more conserved while the substrate pocket is extremely variable, allowing for their functional divergence.

In this scenario, it seems reasonable to propose that class A and class B monooxygenases, which overlap in their taxonomic distribution but not in their reaction profiles, have emerged to solve different problems in nature, converging in their usage of cofactors. Class A monooxygenases are strongly dependent on the presence of substrate in order to enter into the catalytic cycle [63] (step I → II in the catalytic mechanism shown in Fig. 5). In the case of class B monooxygenases, these enzymes are strongly dependent on the presence of NAD(P)H to start the catalytic cycle (step I → II → III in the catalytic cycle). Moreover, when the nicotinamide is present, the reactive intermediate peroxyflavin is formed in the absence of substrate (species IV); thus, class B monooxygenases are ready to start before finding the suitable substrate [64]. It seems that the presence of an extra domain to exclusively recruit the nicotinamide cofactor makes this mechanism possible.

Our analysis of the origin of the diversity and enzyme function of the flavin-dependent monooxygenases demonstrates that a complex set of factors drove the evolution of this family of enzymes. The multidomain organization plays a key role in defining each class and conferring specific properties such as the ability to work with a reductase partner or form multienzymatic complexes. However, the accumulation of point changes on the shared FAD-binding domain has led to the functionalization of each class and, in the case of class B monooxygenases, has ultimately driven the divergence into the three groups of FMOs, BVMOs, and NMOs.

## Materials and Methods

### Datasets, sequences, and PBD codes

Enzymes in the EC sub-subclasses 1.13.13, 1.14.13, and 1.14.14 were identified, analyzed, and collected from the enzyme information system BRENDA† (Table S1). Protein sequences were obtained from the UniProt database‡ (Dataset S1). PDB files were obtained from the RCSB PDB§ [65], while structural information relating to them (e.g., residues interacting with substrates) came from the PDBsum database|| [66] (Table S2).

### Domain architecture analyses

The CATH hierarchical classification of protein domains [29] was employed as the criterion for the analysis of MDA¶. For sequences lacking structural information, their amino acid sequence was submitted to the Gene3D server^a^ and a predicted MDA was retrieved.

### MSAs

Sequence-based MSAs were constructed by employing the MAFFT program version 7 via the online server^b^. Alignments were manually edited to extract the region forming the FAD-binding domain (CATH 3.50.50.60) shared by the flavin‐monooxygenases included in this study.

Structure-based alignments were constructed by employing the tool PDBeFold^c^ [32] for multiple comparison and 3D alignment of protein structures. A list of PDB codes was submitted and the retrieved alignments were manually edited to pull out only those positions that are structurally homologous. All-against-all matrices of Cα-to-Cα RMSD values between the superposed proteins were also obtained to provide measures of their structural similarity.

### Phylogenetic analyses

The initial evolutionary analysis was performed based on the MSAs of the amino acid sequences of the FAD-binding domain regions. The best fit model parameters were calculated using ProtTest version 3.4 [67]. Phylogenetic trees were constructed using the PhyML 3.0 online program^d^ [33]. To estimate the robustness of the phylogenetic inference, we run 100 BS. MEGA 6.0 software was used to visualize and edit the consensus tree.

The structure-based phylogeny employed the 3D alignments from PDBeFold and the same inference method as above. To test the evolutionary hypothesis, we also used Bayesian inference to construct a tree with Mr. Bayes software version 3.2 [68]. The topology of the trees was consistent across the different inference methods.

### 3D structure analyses

Topology diagrams were manually drawn based on the secondary and tertiary structure schemes obtained from PDBsum, as were the residues involved in the binding of ligands.

Structures were visualized, compared, and analyzed using the PyMOL v1.7.6 molecular visualization system. PDB files were obtained from the RCSB PDB. The structure of the separate domains was obtained by manually editing the PDB files according to the information given by CATH.

### Functional divergence of classes

To identify the residues involved in the binding of ligands FAD, NADPH, and substrate, we used the SAS (Sequence Annotated by Structure) server^e^ [47]. The extracted pseudo-sequences of just the interacting residues were used to obtain MSAs from which phylogenetic trees were constructed using the ML inference method as described above. The MEGA 6.0 software was used to visualize and edit the consensus trees.

## Figures and Tables

**Table 1:** Biochemical and structural features of flavin-dependent monooxygenases

Flavin-dependent monooxygenases									
Group	Class	Biochemistry					Structure		
Archetypical enzymes	EC	Cofactor	Hydride donor	Organization	Domain^a^	MDA^b^	PDB^c^
1	A	Aromatic hydroxylases	1.14.13	FAD	NAD(P)H	Single component	3.50.50.60	pA_pX_pA_pX_pA	89 (14)
B	Flavin monooxygenases (FMO) Baeyer–Villiger monooxygenases (BVMO) N-hydroxylating monooxygenases (NMO)	1.14.13	FAD	NADPH	Single component	3.50.50.60	pA_A_pA	36 (6)
E	Epoxidases	1.14.14	FAD	FADH_2_	Two components	3.50.50.60	pA_pX_pA_pX_pA	3 (1)
F	Amino acid halogenases	1.14.14	FAD	FADH_2_	Two components	3.50.50.60	pA_pX_pA_pX_pA_R	9 (3)
G	Amino acid decarboxylases	1.13.12	FAD	Substrate	Single component	3.50.50.60	pA_Q_pA_R_pA	7 (2)
2	C	Luciferases BVMOs type II Hydroxylases	1.14.13	FMN	FMNH_2_	Two components^d^	3.20.20.30	B	2 (1)
3	D	Hydroxylases Epoxidases	1.14.14	FAD/FMN	FADH_2_/FMNH_2_	Two components	1.10.540.10 2.40.110.10	C_D pD_Z_pD	3 (1)
4	H	Decarboxylases Denitrases	1.13.12	FAD	Substrate	Two components	3.20.20.70	E	6 (2)

a The most common CATH domain found within the class is indicated.

b The representative MDA for each class is depicted. A = CATH 3.50.50.60; X = CATH 3.30.9.10; B = CATH 3.20.20.30; C = CATH 1.10.540.10; D = CATH 2.40.110.10; Z = random domain; R = C-terminal domain; Q = CATH 3.90.660.10; and E = CATH 3.20.20.70. The prefix “p” stands for a split domain.

c The number of PBD entries is listed, with the number of different enzymes given in brackets. Data were taken from PDBsum (last accessed on 30th September 2015).

d External reductases

## Appendix A Supplementary Data

Supplementary material Image 2

## Footnotes

† http://www.brenda-enzymes.org/

‡ http://www.uniprot.org/

§ http://www.rcsb.org/pdb/home/home.do

|| http://www.ebi.ac.uk/pdbsum

¶ http://www.cathdb.info/

a http://gene3d.biochem.ucl.ac.uk/

b http://mafft.cbrc.jp/alignment/server/

c http://www.ebi.ac.uk/msd-srv/ssm/

d http://www.atgc-montpellier.fr/phyml/

e http://www.ebi.ac.uk/thornton-srv/databases/sas/
