# Supplementary material for: Chopping and Changing: the Evolution of the Flavin-dependent Monooxygenases
Source: J Mol Biol. 2016 Jul 31;428(15):3131–46. doi: 10.1016/j.jmb.2016.07.003 (PMC4981433; doi:10.1016/j.jmb.2016.07.003)
Supplement: Supplementary file 1 — Supplementary material [file mmc1.pdf]

Chopping and Changing: the Evolution of the Flavin-dependent Monooxygenases

Maria Laura Mascotti[1*], Maximiliano Juri Ayub[1], Nicholas Furnham[2], Janet M. Thornton[3], Roman A Laskowski[3*]

[1]IMIBIO-SL CONICET, Facultad de Química Bioquímica y Farmacia, Universidad Nacional de San Luis, Ejército de los Andes 950, San Luis, D5700HHW, Argentina

[2]Department of Pathogen Molecular Biology, London School of Hygiene and Tropical Medicine, Keppel Street, London, WC1E 7HT, UK

[3]EMBL-EBI, Wellcome Trust Genome Campus, Hinxton, Cambridge, CB10 1SD, UK

[*]corresponding authors:

Maria Laura Mascotti (mlmascotti@unsl.edu.ar), Roman A. Laskowski (roman@ebi.ac.uk)

Supplementary Data

	Page nº
**Table S1**	…………………….. 2-7
**Dataset S1**	………………………. 8
**Table S2**	………………………. 9
**Figure S1**	……………………... 10
**Figure S2**	……………………... 11
**References**	..……………………. 12

Table S1

Classification of enzymes included in the EC sub-subclasses 1.13.12, 1.14.13 and 1.14.14.

Data collected from the enzyme database BRENDA (http://www.brenda-enzymes.org/, last accessed on 8th June 2016). The classes containing flavin-dependent monooxygenases are identified by FMO in the final column and their class (A-H).

EC	PDBs	Enzyme Name	Classification*
1.13.12.1	-	arginine 2-monooxygenase	FMO G
1.13.12.2	-	lysine 2-monooxygenase	FMO G
1.13.12.3	2	tryptophan 2-monooxygenase	FMO G
1.13.12.4	30	lactate 2-monooxygenase	FMO H
1.13.12.5	11	*Renilla*-luciferin 2-monooxygenase	Luc
1.13.12.6	-	*Cypridina*-luciferin 2-monooxygenase	Luc
1.13.12.7	17	*Photinus*-luciferin 4-monooxygenase	Luc
1.13.12.8	-	*Watasenia* luciferin 2-monooxygenase	Luc
1.13.12.9	12	phenylalanine 2-monooxygenase precursor	FMO G
1.13.12.13	-	*Oplophorus*-luciferin 2-monooxygenase catalytic subunit	Luc
1.13.12.15	-	3,4-dihydrophenylalanine oxidative deaminase	-
1.13.12.16	4	nitronate monooxygenase	FMO H
1.13.12.17	-	dichloroarcyrialavin A synthase (RebP)	CYP 450
1.13.12.18	-	dinoflagellate luciferase	Luc
1.13.12.19	-	2-oxoglutarate dioxygenase (ethylene-forming)	Non Heme Fe
1.13.12.20	-	noranthrone monooxygenase	-
1.13.12.21	-	tetracenomycin-F1 monooxygenase	-
1.13.12.22	-	deoxynogalonate monooxygenase	-
1.14.13.1	5	salicylate 1-monooxygenase	FMO A
1.14.13.2	36	4-hydroxybenzoate 3-monooxygenase (NADPH)	FMO A
1.14.13.3	-	4-hydroxyphenylacetate 3-monooxygenase	FMO
1.14.13.4	-	Melilotate 3-monooxygenase	FMO
1.14.13.5	-	imidazoleacetate 4-monooxygenase	FMO A
1.14.13.6	-	orcinol 2-monooxygenase (OrcA)	FMO A
1.14.13.7	9	phenol 2-monooxygenase	FMO A
1.14.13.8	48	dimethylaniline monooxygenase (mammalian FMO)	FMO B

1.14.13.9	10	kynurenine 3-monooxygenase	FMO A
1.14.13.10	3	2,6-dihydroxypyridine 3-monooxygenase	FMO A
1.14.13.11	-	trans-cinnamate 4-monooxygenase	CYP 450
1.14.13.12	-	benzoate 4-monooxygenase	CYP 450
1.14.13.13	-	calcidiol 1-monooxygenase	CYP 450
1.14.13.14	-	trans-cinnamate 2-monooxygenase	CYP 450
1.14.13.15	18	cholestanetriol 26-monooxygenase	CYP 450
1.14.13.16	-	cyclopentanone monooxygenase	FMO B
1.14.13.17	6	cholesterol 7α-monooxygenase	CYP 450
1.14.13.18	-	4-hydroxyphenylacetate 1-monooxygenase	FMO A
1.14.13.19	-	taxifolin 8-monooxygenase	FMO
1.14.13.20	-	2,4-dichlorophenol 6-monooxygenase	FMO A
1.14.13.21	-	flavonoid 3'-monooxygenase	CYP 450
1.14.13.22	5	cyclohexanone monooxygenase	FMO B
1.14.13.23	2	3-hydroxybenzoate 4-monooxygenase	FMO A
1.14.13.24	5	3-hydroxybenzoate 6-monooxygenase	FMO A
1.14.13.25	180	methane monooxygenase (soluble)	FMO
1.14.13.27	-	4-aminobenzoate 1-monooxygenase	FMO A
1.14.13.28	-	3,9-dihydroxypterocarpan 6a-monooxygenase	CYP 450
1.14.13.29	-	4-nitrophenol 2-monooxygenase	FMO A
1.14.13.30	-	leukotriene-B4 20-monooxygenase	CYP 450
1.14.13.31	-	2-nitrophenol 2-monooxygenase	FMO A
1.14.13.32	-	albendazole monooxygenase	CYP 450
1.14.13.33	-	4-hydroxybenzoate 3-monooxygenase	FMO A
1.14.13.34		leukotriene-E4 20-monooxygenase	CYP 450
1.14.13.35	-	anthranilate 3-monooxygenase (deaminating)	Non Heme Fe
1.14.13.36	-	5-O-(4-coumaroyl)-D-quinate 3′-monooxygenase	FMO
1.14.13.37	-	methyltetrahydroprotoberberine 14-monooxygenase	CYP 450
1.14.13.38	-	anhydrotetracycline monooxygenase	FMO A
1.14.13.39	792	nitric-oxide synthase (NADPH dependent)	Reductase: FAD/FMN/Fe-S<br>Oxidase: HEME + THBP
1.14.13.40	-	anthraniloyl-CoA monooxygenase	FMO A
1.14.13.41	-	tyrosine N-monooxygenase	CYP 450
1.14.13.43	-	questin monooxygenase	unknown
1.14.13.44	8	2-hydroxybiphenyl 3-monooxygenase	FMO A
1.14.13.46	-	(-)-menthol monooxygenase	CYP 450
1.14.13.47	-	(S)-limonene 3-monooxygenase	CYP 450
1.14.13.48	18	(S)-limonene 6-monooxygenase	CYP 450

1.14.13.49	-	(S)-limonene 7-monooxygenase	CYP 450
1.14.13.50	-	pentachlorophenol monooxygenase	FMO A
1.14.13.51	-	6-oxocineole dehydrogenase	unknown
1.14.13.52	-	isoflavone 3'-hydroxylase	CYP 450
1.14.13.53	-	4′-methoxyisoflavone 2′-hydroxylase	CYP 450
1.14.13.54	4	ketosteroid monooxygenase	FMO B
1.14.13.55	-	protopine 6-monooxygenase	CYP 450
1.14.13.56	-	dihydrosanguinarine 10-monooxygenase	CYP 450
1.14.13.57	-	dihydrochelirubine 12-monooxygenase	CYP 450
1.14.13.58	-	benzoyl-CoA 3-monooxygenase	FMO A
1.14.13.59	-	L-lysine N6-monooxygenase (NADPH)	FMO B
1.14.13.61	18	2-hydroxyquinoline 8-monooxygenase	Non Heme Fe
1.14.13.62	-	4-hydroxyquinoline 3-monooxygenase	unknown
1.14.13.63	-	3-hydroxyphenylacetate 6-hydroxylase	FMO A
1.14.13.64	-	4-hydroxybenzoate 1-hydroxylase	FMO A
1.14.13.66	-	2-hydroxycyclohexanone 2-monooxygenase	unknown
1.14.13.67	-	quinine 3-monooxygenase	CYP 450
1.14.13.68	-	4-hydroxyphenylacetaldehyde oxime monooxygenase	CYP 450
1.14.13.69	-	alkene monooxygenase	Non Heme Fe
1.14.13.70	85	sterol 14α-demethylase	CYP 450
1.14.13.71	-	N-methylcoclaurine 3'-monooxygenase	CYP 450
1.14.13.72	-	methylsterol monooxygenase	CYP B5
1.14.13.73	-	tabersonine 16-hydroxylase	CYP 450
1.14.13.76	-	taxane 10β-hydroxylase	CYP 450
1.14.13.77	-	taxane 13α-hydroxylase	CYP 450
1.14.13.78	-	ent-kaurene oxidase	CYP 450
1.14.13.79	-	ent-kaurenoic acid oxidase	CYP 450
1.14.13.80	26	(R)-limonene 6-monooxygenase	-
1.14.13.81	-	magnesium-protoporphyrin IX monomethyl ester (oxidative) cyclase	Non Heme Fe
1.14.13.82	-	vanillate monooxygenase	Non Heme Fe
1.14.13.83	-	precorrin-3B synthase	Fe-S
1.14.13.84	-	4-hydroxyacetophenone monooxygenase	FMO B
1.14.13.85	-	glyceollin synthase	CYP 450
1.14.13.86	-	2-hydroxyisoflavanone synthase	CYP 450
1.14.13.87	-	licodione synthase	CYP 450
1.14.13.88	-	flavanoid 3',5'-hydroxylase	CYP 450
1.14.13.89	-	isoflavone 2'-hydroxylase	CYP 450
1.14.13.90	-	zeaxanthin epoxidase	FMO E(A)

1.14.13.91	-	deoxysarpagine hydroxylase	CYP 450
1.14.13.92	14	phenylacetone monooxygenase	FMO B
1.14.13.93	-	(+)-abscisic acid 8'-hydroxylase	CYP 450
1.14.13.94	-	lithocholate 6β-hydroxylase	CYP 450
1.14.13.95	-	7α-hydroxycholest-4-en-3-one 12α-hydroxylase	CYP 450
1.14.13.96	-	5β-cholestane-3α,7α-diol 12α-hydroxylase	CYP 450
1.14.13.97	-	taurochenodeoxycholate 6α-hydroxylase	CYP 450
1.14.13.98	12	cholesterol 24-hydroxylase	CYP 450
1.14.13.99	-	24-hydroxycholesterol 7α-hydroxylase	CYP 450
1.14.13.100	-	25/26-hydroxycholesterol 7α-hydroxylase	CYP 450
1.14.13.101	-	senecionine N-oxygenase	FMO B
1.14.13.102	-	psoralen synthase	CYP 450
1.14.13.103	-	8-dimethylallylnaringenin 2′-hydroxylase	CYP 450
1.14.13.104	-	(+)-menthofuran synthase	CYP 450
1.14.13.105	-	monocyclic monoterpene ketone monooxygenase	FMO B
1.14.13.106	4	epi-isozizaene 5-monooxygenase	CYP 450
1.14.13.107	-	limonene 1,2-monooxygenase	FMO E(A)
1.14.13.108	-	abieta-7,13-diene hydroxylase	CYP 450
1.14.13.109	-	abieta-7,13-dien-18-ol hydroxylase	CYP 450
1.14.13.110	-	geranylgeraniol 18-hydroxylase	CYP 450
1.14.13.111	-	methanesulfonate monooxygenase	FMO
1.14.13.112	-	3-epi-6-deoxocathasterone 23-monooxygenase	CYP 450
1.14.13.113	3	FAD-dependent urate hydroxylase	FMO A
1.14.13.114	-	6-hydroxynicotinate 3-monooxygenase	FMO A
1.14.13.115	-	angelicin synthase	CYP 450
1.14.13.116	-	geranylhydroquinone 3′′-hydroxylase	CYP 450
1.14.13.117	-	isoleucine N-monooxygenase	CYP 450
1.14.13.118	-	valine N-monooxygenase	CYP 450
1.14.13.119	-	5-epiaristolochene 1,3-dihydroxylase	CYP 450
1.14.13.120	-	costunolide synthase	CYP 450
1.14.13.121	-	premnaspirodiene oxygenase	CYP 450
1.14.13.122	-	chlorophyllide-a oxygenase	Non Heme Fe
1.14.13.123	-	germacrene A hydroxylase	CYP 450
1.14.13.124	-	phenylalanine N-monooxygenase	CYP 450
1.14.13.125	-	tryptophan N-monooxygenase	CYP 450
1.14.13.126	4	vitamin D3 24-hydroxylase	CYP 450
1.14.13.127	-	3-(3-hydroxyphenyl)propanoate hydroxylase	FMO A
1.14.13.128	-	7-methylxanthine demethylase	Non Heme Fe

1.14.13.129	-	beta-carotene 3-hydroxylase	Non Heme Fe
1.14.13.130	-	pyrrole-2-carboxylate monooxygenase	FMO
1.14.13.131	-	dimethyl-sulfide monooxygenase	FMO C
1.14.13.132	-	squalene monooxygenase	FMO E(A)
1.14.13.133	-	pentalenene oxygenase	CYP 450
1.14.13.134	-	β-amyrin 11-oxidase	CYP 450
1.14.13.135	-	1-hydroxy-2-naphthoate hydroxylase	unknown
1.14.13.136	-	2-hydroxyisoflavanone synthase	CYP 450
1.14.13.137	-	indole-2-monooxygenase	CYP 450
1.14.13.138	-	indolin-2-one monooxygenase	CYP 450
1.14.13.139	-	3-hydroxyindolin-2-one monooxygenase	CYP 450
1.14.13.140	-	2-hydroxy-1,4-benzoxazin-3-one monooxygenase	CYP 450
1.14.13.141	8	cholest-4-en-3-one 26-monooxygenase	CYP 450
1.14.13.142	2	3-ketosteroid 9alpha-monooxygenase	Non Heme Fe
1.14.13.143	-	ent-isokaurene C2-hydroxylase	CYP 450
1.14.13.144	-	9β-pimara-7,15-diene oxidase	CYP 450
1.14.13.145	-	ent-cassa-12,15-diene 11-hydroxylase	CYP 450
1.14.13.146	-	taxoid 14beta-hydroxylase	CYP 450
1.14.13.147	-	taxoid 7beta-hydroxylase	CYP 450
1.14.13.148	-	trimethylamine monooxygenase	FMO B
1.14.13.149	15	phenylacetyl-CoA 1,2-epoxidase	FMO
1.14.13.150	-	α-humulene 10-hydroxylase	CYP 450
1.14.13.151	-	linalool 8-monooxygenase	CYP 450
1.14.13.152	-	geraniol 8-hydroxylase	CYP 450
1.14.13.153	-	(+)-sabinene 3-hydroxylase	CYP 450
1.14.13.154	6	erythromycin 12-hydroxylase	CYP 450
1.14.13.155	-	α-pinene monooxygenase	CYP 450
1.14.13.156	20	1,8-cineole 2-endo-monooxygenase	CYP 450
1.14.13.157	-	1,8-cineole 2-exo-monooxygenase	CYP 450
1.14.13.158	-	amorpha-4,11-diene 12-monooxygenase	CYP 450
1.14.13.159	6	vitamin D 25-hydroxylase	CYP 450
1.14.13.160	12	(2,2,3-trimethyl-5-oxocyclopent-3-enyl)acetyl-CoA 1,5-monooxygenase	FMO B
1.14.13.161	-	(+)-camphor 6-exo-hydroxylase	CYP 450
1.14.13.162	2	2,5-diketocamphane 1,2-monooxygenase	FMO C
1.14.13.163	-	6-hydroxy-3-succinoylpyridine 3-monooxygenase	FMO A
1.14.13.165	24	nitric-oxide synthase [NAD(P)H-dependent]	Reductase: FAD/FMN/Fe-S<br>Oxidase: Heme + THBP
1.14.13.166	-	4-nitrocatechol 4-monooxygenase	FMO D

1.14.13.167	-	4-nitrophenol 4-monooxygenase	FMO
1.14.13.168	-	indole-3-pyruvate monooxygenase	FMO B
1.14.13.169	-	sphinganine C4-monooxygenase	Fe
1.14.13.170	-	pentalenolactone D synthase	FMO B
1.14.13.171	-	neopentalenolactone D synthase	FMO B
1.14.13.172	-	salicylate 5-hydroxylase	Fe-S
1.14.13.173	-	11-oxo-beta-amyrin 30-oxidase	CYP 450
1.14.13.174	-	averantin hydroxylase	CYP 450
1.14.13.175	-	aflatoxin B synthase	CYP 450
1.14.13.176	-	tryprostatin B 6-hydroxylase	CYP 450
1.14.13.177	-	fumitremorgin C monooxygenase	CYP 450
1.14.13.178	-	methylxanthine N1-demethylase	Non Heme Fe
1.14.13.179	-	methylxanthine N3-demethylase	Non Heme Fe
1.14.13.180	5	aklavinone 12-hydroxylase	FMO
1.14.13.181	-	13-deoxydaunorubicin hydroxylase	CYP 450
1.14.13.182	-	2-heptyl-3-hydroxy-4(1H)-quinolone synthase	FMO
1.14.13.183	-	dammarenediol 12-hydroxylase	CYP 450
1.14.13.184	-	protopanaxadiol 6-hydroxylase	CYP 450
1.14.13.185	41	pikromycin synthase	CYP 450
1.14.13.186	-	20-oxo-5-O-mycaminosyltylactone 23-monooxygenase	CYP 450
1.14.13.187	-	L-evernosamine nitrososynthase	FMO
1.14.13.188	9	6-deoxyerythronolide B hydroxylase	CYP 450
1.14.13.189	-	5-methyl-1-naphthoate 3-hydroxylase	CYP 450
1.14.13.190	-	ferruginol synthase (CYP76AH1)	CYP 450
1.14.13.191	-	ent-sandaracopimaradiene 3-hydroxylase	CYP 450
1.14.13.192	-	oryzalexin E synthase	CYP 450
1.14.13.193	-	oryzalexin D synthase	CYP 450
1.14.13.194	-	phylloquinone ω-hydroxylase	CYP 450
1.14.13.195	4	L-ornithine N5-monooxygenase (NADPH)	FMO B
1.14.13.196	8	L-ornithine N5-monooxygenase [NAD(P)H]	FMO B
1.14.13.197	-	dihydromonacolin L hydroxylase	CYP 450
1.14.13.198	-	monacolin L hydroxylase	CYP 450
1.14.13.199	-	docosahexaenoic acid omega-hydroxylase	CYP 450
1.14.13.200	-	tetracenomycin A2 monooxygenase-dioxygenase	FMO
1.14.13.201	-	β-amyrin 28-monooxygenase	CYP 450
1.14.13.202	-	methyl farnesoate epoxidase	CYP 450
1.14.13.203	-	farnesoate epoxidase	CYP 450
1.14.13.204	-	long-chain acyl-CoA ω-monooxygenase	CYP 450

1.14.13.205	-	long-chain fatty acid ω-monooxygenase	CYP 450
1.14.14.1	23	cytochrome P450 1A2	CYP 450
1.14.14.3	14	alkanal monooxygenase FMN	FMO C
1.14.14.5	3	alkanesulfonate monooxygenase	FMO C
1.14.14.7	-	tryptophan 7-halogenase	FMO F
1.14.14.8	-	anthranilate 3-monooxygenase oxygenase component	FMO D
1.14.14.9	23	p-hydroxyphenylacetate 3-hydroxylase, oxygenase component	FMO D
1.14.14.10	-	nitrilotriacetate monooxygenase component A	FMO C
1.14.14.11	-	styrene monooxygenase StyA	FMO E
1.14.14.12	6	flavin-dependent monooxygenase, oxygenase subunit HsaA	Reductase: FAD/FMN-Oxygenase
1.14.14.13	-	4-(gamma-L-glutamylamino)butanoyl monooxygenase	-
1.14.14.14	6	aromatase	CYP 450
1.14.14.15	-	(3S)-3-amino-3-(3-chloro-4-hydroxyphenyl)propanoyl monooxygenase	FMO
1.14.14.x	1	alkylhalidase CmlS	FMO F
1.14.14.x	3	tryptophan 5-halogenase	FMO F
1.14.14.x	5	tryptophan 7-halogenase	FMO F

* The classification is based on the biochemistry performed by the enzyme. For flavin-dependent monooxygenases a detailed classification according to Huijbers, *et al*[1] is also included.

References:

FMO: flavin-dependent monooxygenase; A-H: classes according to Huijbers, *et al*.

CYP: cytrochrome

Fe-S: iron sulfur proteins

Non Heme Fe: proteins dependent on Fe^{+2} or containing the Rieske active site [2Fe-2S] (ferredoxin) or NAD(P)H dependent or multi-component enzymes.

Luc: luciferase

Dataset S1

UniProt codes of enzymes included in this study belonging to the EC sub-subclasses 1.13.12, 1.14.13 and 1.14.14.

D7I5Z1	Q04564	Q9HDX2	P27652	P17554	P08659	Q5W9R9
Q9GV45	P47177	O77206	P32021	B8NI03	P39889	P23262
P00438	Q7CHV9	C4WK13	Q7WTJ6	P31513	P38169	Q93NG3
P92994	P17549	O15528	Q02318	Q8GAW0	P22680	E5AKV1
P27138	Q9SD85	P12015	Q6SSJ6	Q8NLB6	P18798	Q92402
Q42798	Q6F4M8	P78329	A7KS54	P08684	D8ISN8	D8SCG3
L7X3S1	Q58PK7	P29473	A9I1N7	Q43135	Q9XHE6	Q8Z8H2
P33261	P42535	Q6WNQ9	Q6WNR0	F2Z9C1	B3R4P7	P11295
A1K6X2	Q078T0	P08684	O48958	Q16850	O64899	P53045
P98183	Q9AXM6	Q8W4T9	Q5Z5R4	Q9C5Y2	P33261	Q9M591
P12609	P21637	Q93TJ5	K9JZ57	P93149	P48418	P93147
Q9FGC7	Q47PU3	Q6ZDE3	Q64148	Q9UNU6	Q7YRB2	P08684
Q9Y6A2	Q9NYL5	O75881	Q8MP06	C0SJS4	Q947B7	P82679
Q9K498	Q9EUT9	Q50EK6	Q9X404	Q9M066	A6T923	Q88FY2
C0SJS3	Q6J540	Q9M7B8	Q94FM7	G3GBK0	A6YIH8	Q9MBA1
D5JBW8	Q9FLC8	O81346	Q09128	P77397	F0E1K6	Q9LTG0
E9JFX9	P32476	Q82IY3	B5BSX1	Q9SXS3	Q43257	Q43255
Q43250	P9WPP1	P71875	A3A871	Q0JF01	Q6YTF1	Q84KI1
Q6JTJ0	P31513	P76077	E3W9C4	Q59723	Q8VWZ7	P48635
Q8VQF6	P08684	Q1PS23	Q6VVX0	H3JQW0	Q6STM1	B1N1A2
O34453	Q6F4M8	C1I201	Q9SVQ1	Q9AST3	E3VWK3	Q82IY8
O52379	H1A981	Q12732	O13345	Q4WAW5	B9WZX6	H9N289
H9N290	Q54530	Q59971	Q02N79	H2DH16	I7CT85	O87605
Q9ZHQ1	Q00441	B4XY99	S4UX02	Q0DBF4	Q6YTF5	Q6YTF1
P78329	Q51548	E9QYP0	Q9Y7C8	Q9Y7C8	Q08477	P04799
P07740	B6VM19	P80645	G7FY55	A4IT51	Q6Q272	P54989
O06834	P9WJA1	Q4H4E5	P11511	P95480	Q0VZ69	A4D0H5
A1E280	Q63KH5	Q9AL91	Q9X3R1	O87009	F8GWP8	B3TMR1

Table S2

PDB structures used in this work.

PDB	Enzyme Name	Chain	FAD	NADPH	Substrate
**Class A monooxygenases**					
1K0J	*p*-hydroxybenzoate hydroxylase	A	✓	NAP (NADPH)	-
1PBE	*p*-hydroxybenzoate hydroxylase	A	✓	-	PHB (p-Hydroxybenzoic acid)
2VOU	2,6-dihydroxypyridine hydroxylase	A	✓	-	-
3RP6	FAD-dependent urate oxidase (HpxO)	A	✓	-	-
4BJZ	3-hydroxybenzoate 6-hydroxylase	A	✓	-	-
2RGJ	Pyocyanin biosynthetic protein (PhzS)	A	✓	-	-
4A6N	Tetracycline-degrading monooxygenase (TetX2)	A	✓	-	T1C (Tigecycline)
4K22	2-octaprenylphenol hydroxylase (UbiI)	A	-	-	-
4J31	Kynurenine 3-monooxygenase	A	✓	-	-
2BRY	MICAL	A	✓	-	-
3IHG	Aklavinone 12-hydroxylase (RdmE)	A	✓	-	VAK (Aklavinone)
2QA1	Angucycline hydroxylase (PgaE)	A	✓	-	-
2R0P	Rebeccamycin monooxygenase (RebC)	A	✓	-	K2C (6,7,12,13-Tetrahydro-5h-Indolo[2,3-A]pyrrolo[3,4- C]carbazol-5-One)
1FOH	Phenol 2-monooxygenase	A	✓	-	IPH (phenol)
2DKH	3-hydroxybenzoate 4-monooxygenase	A	✓	-	3HB (3-Hydroxybenzoic acid)
	**CLASS F MONOOXYGENASES**				
3I3L	Alkylhalidase (CmlS)	A	✓	-	-
2WEU	Tryptophan 5-halogenase	A	-	-	TRP (tryptophan)
2AQJ	Tryptophan 7-halogenase	A	✓	-	TRP (tryptophan)
	**CLASS E MONOOXYGENASES**				
3IHM	Styrene monooxygenase (oxygenase component)	A	-	-	-
	**CLASS G MONOOXYGENASES**				
4IV9	Tryptophan 2-monooxygenase	A	✓	-	TSR (2-(1h-Indol-3-Yl)acetamide)
3AYI	Phenylalanine 2-monooxygenase	A	✓	-	-
	**CLASS B MONOOXYGENASES**				
2VQ7	FMO from *Methylophaga* sp.	A	✓	NAP (NADPH)	-
2GV8	FMO from *Schizosaccharomyces pombe*	A	✓	NAP (NADPH)	-
3S5W	Ornithine hydroxylase	A	✓	NAP (NADPH)	ONH (N-5-Hydroxy-L-ornithine)
4B63	L-ornithine N(5)-monooxygenase (SidA)	A	✓	NAP (NADPH)	ORN (L-ornithine)
4AOS	Steroid monooxygenase (STMO)	A	✓	NAP (NADPH)	-
3GWF	Cyclohexanone monooxygenase (CHMO)	A	✓	NAP (NADPH)	-
2YLT	Phenylacetone monooxygenase (PAMO)	A	✓	NAP (NADPH)	MES (2-(N-Morpholino)-ethanesulfonic acid)

3UOZ	2-oxo-Delta(3)-4,5,5-trimethylcyclopentenylacetyl-CoA monooxygenase (OTEMO)	A	✓	NAP (NADPH)	-

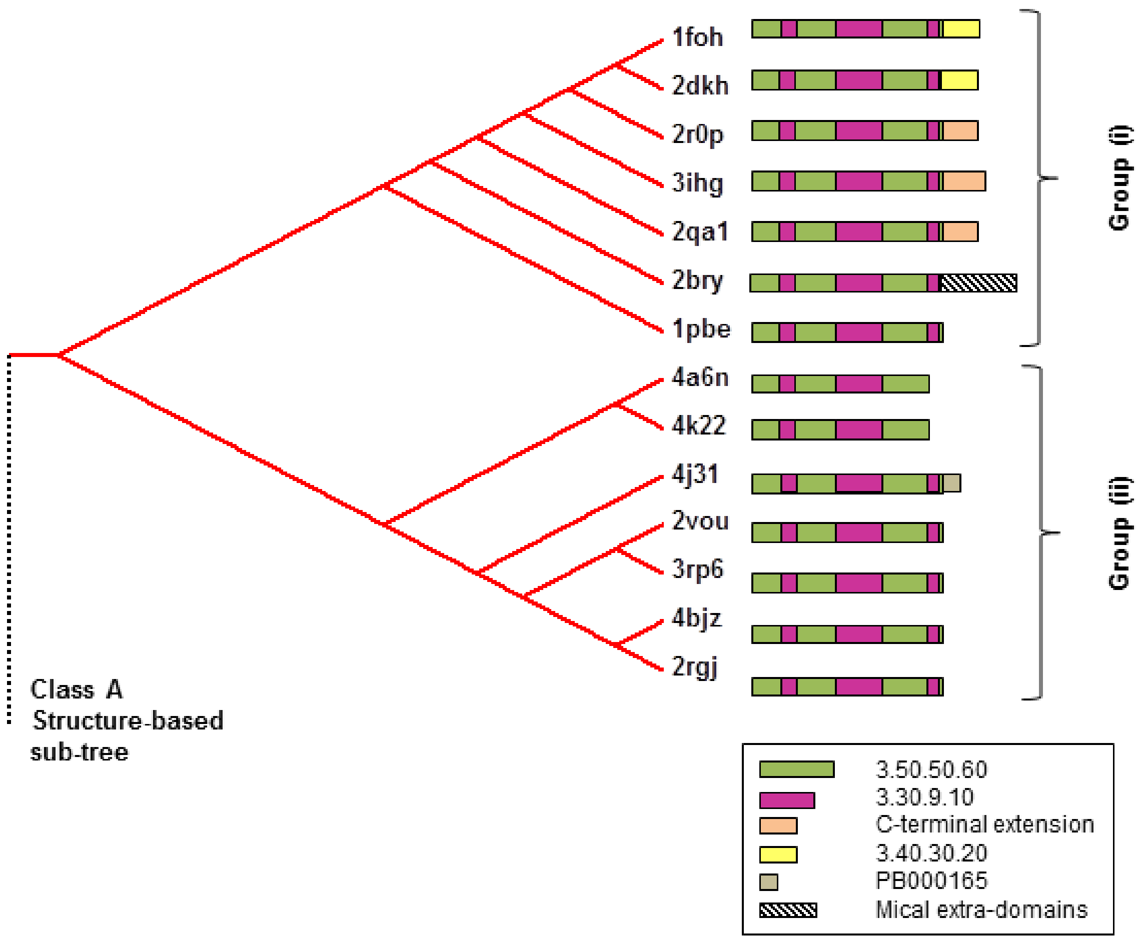

Figure S1: Structure-based sub tree of flavin-dependent monooxygenases belonging to class A.

The sub tree shown here comes from the tree in Fig.1, and has the MDA of each structure shown by the colored bars on the right. Each color corresponds to a specific CATH domain, as identified by the key at bottom right. Note that the green and purple domains (3.50.50.60 and 3.30.9.10) are both 'split' domains consisting of non-contiguous amino acid sequences. Each protein contains a single copy of both these domains, split into 3 or 4 parts.

The clustering into groups (i) and (ii) account for the modifications in the domain architecture observed in each structure. Proteins in group (i) all contain variable C-terminal domains connected to the basic MDA represented by the structure **1PBE**. Proteins contained in group (ii) are less decorated in the C-terminal region. The basal architecture in this case is represented by structures **4A6N** and **4K22**.

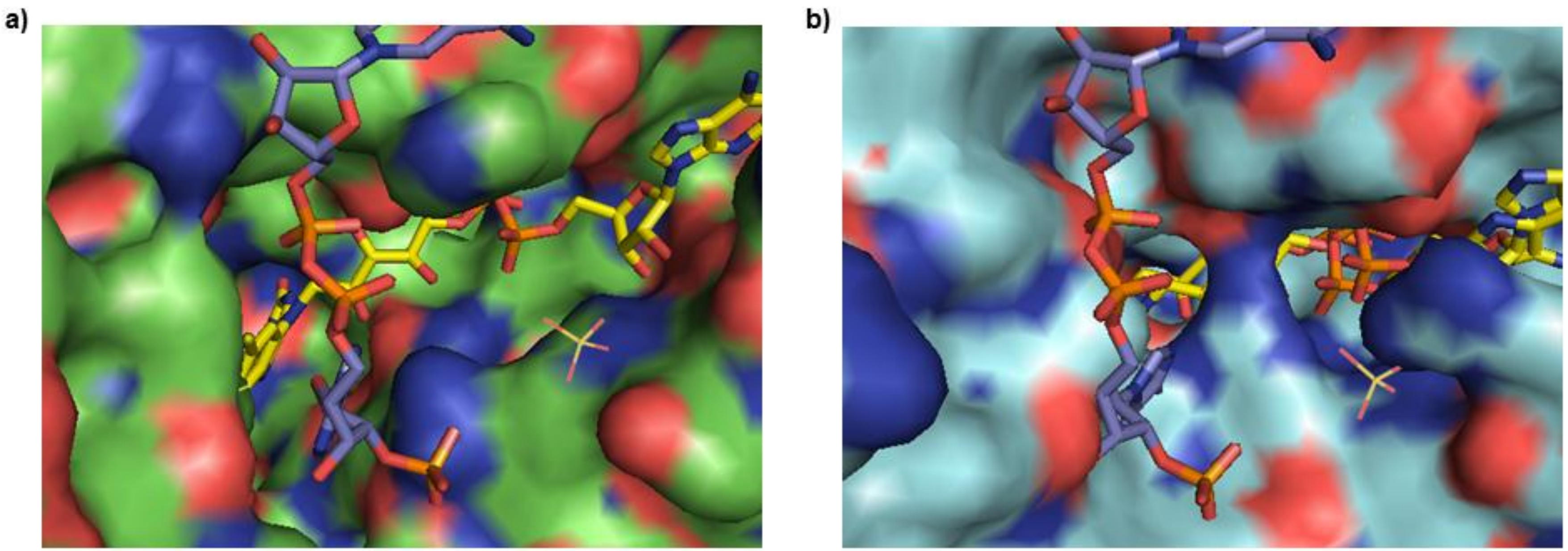

Figure S2: Class A binding of NADPH.

a) The image shows the binding of NADPH (mainly blue molecule in stick representation at the front) in a groove in the surface of a class A monooxygenase (PDB code: **1K0J**). FAD (mainly yellow molecule in stick representation at the back) binding site is also depicted[2]. **b)** The 'simulated binding' of NADPH to the surface of a class E monooxygenase (PDB code: **3IHM**) demonstrates that NADPH is unable to bind here due to the absence of the groove in the surface. Therefore, this class, as well as class F, needs a reductase partner to provide the reduced flavin[3; 4].

References

1. Huijbers, M. M., Montersino, S., Westphal, A. H., Tischler, D. & van Berkel, W. J. (2014). Flavin dependent monooxygenases. *Arch Biochem Biophys* **544**, 2-17.
2. Wang, J., Ortiz-Maldonado, M., Entsch, B., Massey, V., Ballou, D. & Gatti, D. L. (2002). Protein and ligand dynamics in 4-hydroxybenzoate hydroxylase. *Proceedings of the National Academy of Sciences* **99**, 608-613.
3. Ukaegbu, U. E., Kantz, A., Beaton, M., Gassner, G. T. & Rosenzweig, A. C. (2010). Structure and ligand binding properties of the epoxidase component of styrene monooxygenase. *Biochemistry* **49**, 1678-88.
4. Montersino, S., Tischler, D., Gassner, G. T. & van Berkel, W. J. H. (2011). Catalytic and Structural Features of Flavoprotein Hydroxylases and Epoxidases. *Advanced Synthesis & Catalysis* **353**, 2301-2319.
